# Urinary Extracellular Vesicles and Their miRNA Cargo in Patients with Fabry Nephropathy

**DOI:** 10.3390/genes12071057

**Published:** 2021-07-09

**Authors:** Tina Levstek, Teo Mlinšek, Marija Holcar, Katja Goričar, Metka Lenassi, Vita Dolžan, Bojan Vujkovac, Katarina Trebušak Podkrajšek

**Affiliations:** 1 Institute of Biochemistry and Molecular Genetics, Faculty of Medicine, University of Ljubljana, Vrazov trg 2, 1000 Ljubljana, Slovenia; tina.levstek@mf.uni-lj.si (T.L.); tm5877@student.uni-lj.si (T.M.); marija.holcar@mf.uni-lj.si (M.H.); katja.goricar@mf.uni-lj.si (K.G.); metka.lenassi@mf.uni-lj.si (M.L.); vita.dolzan@mf.uni-lj.si (V.D.); 2 Centre for Fabry Disease, General Hospital Slovenj Gradec, Gosposvetska cesta 1, 2380 Slovenj Gradec, Slovenia; bojan.vujkovac@sb-sg.si; 3 Clinical Institute for Special Laboratory Diagnostics, University Children’s Hospital, University Medical Centre Ljubljana, Vrazov trg 1, 1000 Ljubljana, Slovenia

**Keywords:** Fabry nephropathy, urinary extracellular vesicles, biomarker, miRNA expression, NTA, Fabry disease, lysosomal storage disease

## Abstract

Current biomarkers of Fabry nephropathy lack sensitivity in detecting early kidney damage and do not predict progression of nephropathy. Urinary extracellular vesicles (uEVs) and their molecular cargo could reflect early changes in renal impairment as they are secreted by the cells lining the urinary tract. We aimed to conduct a proof-of-concept study to investigate whether analysis of uEV characteristics and expression of uEV-derived microRNAs (miRNAs) could be applicable in studies to predict the development and progression of nephropathy in Fabry disease. A total of 20 Fabry patients were divided into two groups, depending on the presence of nephropathy. Chronological urine samples collected during 10-year follow-up were used for uEVs isolation with size exclusion chromatography. Nanoparticle tracking analysis was used to determine concentration and size of uEVs. We evaluated the expression of five uEV-derived miRNAs by qPCR (miR-23a-3p, miR-29a-3p, miR-30b-5p, miR-34a-5p, miR-200a-3p). There was no difference in the concentration and size of uEVs between patients with and without nephropathy at last follow-up or longitudinally. However, we found increased expression of miR-29a-3p and miR-200a-3p in uEVs isolated from chronological samples of patients with Fabry nephropathy. This may indicate an attempt by the organism to prevent the progression of renal damage leading to end-stage renal disease as previously reported in type 1 diabetes. In addition, we found an increased expression of miR-30b-5p in the 10-year period in uEVs of patients without renal dysfunction. miR-30b-5 was reported to have a protective role in podocyte injury and may possibly be important in Fabry nephropathy. These findings indicate that uEVs and their molecular cargo could be a promising target of studies focusing on elucidation of Fabry nephropathy. Nevertheless, total concentration and size of uEVs were neither indicative of the presence nor progression of Fabry nephropathy, while the role of the analyzed miRNAs in Fabry nephropathy progression was merely indicated and needs further in-depth studies.

## 1. Introduction

Fabry disease (FD; OMIM ID #301500) is a rare lysosomal genetic disorder characterized by a deficiency of α-galactosidase A (α-gal A) [1], which leads to the accumulation of globotriaosylceramide (Gb3), globotriaosylsphingosine (lyso-Gb3), and other glycosphingolipids in various cell types throughout the body [2]. With advancing age, this can lead to various life-threatening conditions, such as cardiac, cerebrovascular, and renal events [3]. Fabry nephropathy contributes significantly to the morbidity and mortality associated with FD [4]. Renal dysfunction usually begins in the second to third decade of life, and is characterized by albuminuria, followed by the development of overt proteinuria, which directly contribute to the progression of Fabry nephropathy [3]. The metabolic origin and rate of progression is similar to that of diabetic nephropathy, and untreated FD patients develop end-stage renal disease by the age of 50 [3,4,5]. In addition, FD patients with Fabry nephropathy are also at higher risk for dysfunction of other organ systems [6].

Timely initiation of treatment is a paramount for stabilizing renal function or at least slowing its decline [7]. Due to the lack of specific clinical markers, diagnosis is often delayed, which compromises the effectiveness of enzyme replacement therapy (ERT) [8]. Indeed, in patients with overt proteinuria or reduced glomerular filtration rate (GFR), ERT does not prevent further deterioration of renal function [8]. Currently used markers of renal function, such as albuminuria, proteinuria, and estimated or measured GFR (eGFR or mGFR) are late markers of renal impairment [9]. Moreover, there is high variability in age of disease onset and disease progression even between the members of the same family [10]. Therefore, there is an urgent need to identify markers that will enable the identification of patients at higher risk for developing or more rapidly progressing Fabry nephropathy (reviewed in [9]), which will lead to a better outcome for the patients.

Extracellular vesicles (EVs) are heterogeneous particles enclosed in a membrane bilayer that function as intercellular communicators by transporting their cargo, which includes nucleic acids, proteins, lipids, and metabolites, to target cells [11]. Urinary EVs (uEVs) originate from cells of all segments of the nephron and collecting duct [12,13]. Thus, uEVs can mediate cellular communication across different regions of the nephron and even between glomerular and tubular cells [14]. The effect of EVs mainly depends on the patho/physiological state of their site of origin; EVs originating from the cells with regenerative potential promote tissue regeneration, while the cells originating from injured cells accelerate disease progression, inflammation, and fibrosis [15,16]. Therefore, uEVs are investigated as potential non-invasive biomarkers for various renal diseases (reviewed in [17]). Recently, EVs also showed a great potential as vesicles for the delivery of α-gal A in FD patients. Namely, EVs restored lysosomal function more efficiently compared to ERT, and were taken up in all tissues, including the brain [18].

uEVs also contain microRNAs (miRNAs), which are small non-coding RNAs involved in negative post-transcriptional regulation by degradation or repression of target messenger RNA (mRNA) [19]. Because vesicular membrane protects miRNAs from degradation by RNases, uEV-derived miRNAs are more stable compared to cell-free urinary miRNAs [20]. miRNAs have been demonstrated to play an important role in kidney development, maintenance of kidney function, and progression of renal dysfunction [21]. In addition, altered expressions of several miRNAs, which reflect the pathophysiological processes, have been detected [22,23].

Since urine forms in the kidney and can be collected non-invasively in large quantities, uEVs and their molecular cargo seem promising for the discovery of early biomarkers of renal injury. To our knowledge, uEVs have not been studied in FD patients so far. The objective of the present study was to conduct proof-of-concept study to investigate whether analysis of uEVs characteristics and uEV-derived miRNAs could be applicable in studies to predict the development and progression of nephropathy in patients with FD. 

## 2. Materials and Methods

### 2.1. Study Subjects

Twenty participants were recruited from the Slovenian national Centre for Fabry disease in General Hospital Slovenj Gradec. The study protocol was approved by the Slovenian Ethics Committee for Research in Medicine (KME 0120-260/2020/6 and KME 0120-521/2020/3). All participants gave written informed consent in accordance with the Declaration of Helsinki. Inclusion criteria were age over 18 years and genetically confirmed FD. The exclusion criteria were physical or psychological disease that might interfere with the normal conduct of the study, pregnancy at the time of enrolment or within the past 12 months, and current or recent history of alcohol or drug abuse. In addition, we excluded patients with uncontrolled hypertension, additional glomerular diseases, and patients on dialysis. Clinical and laboratory examinations were performed as already routinely scheduled. Renal function was estimated using the Chronic Kidney Disease Epidemiology Collaboration (CKD-EPI) equation [24].

Second morning urine was collected for isolation of uEVs. Urine was collected and kept on ice before further processing. Urine samples were centrifuged at 2000× *g* for 15 min within 3 h to remove cells and debris, after which the supernatant was transferred to new tubes and stored at −80 °C. In addition, periodic urine samples from individual patients were already collected during their previous routine check-ups and stored at −80 °C. Periodic samples were collected in the last 10 years at approximately every 5 years. A total of 52 samples from 20 participants were assessed. A cohort of 18 patients was analyzed for the 5-year period, while 14 patients were included for the 10-year period, as some patients were diagnosed within the last 10 years.

Participants were divided into two cohorts: (1) subjects with Fabry nephropathy consisting of patients with present albuminuria with urinary albumin-to-creatinine ratio (UACR) > 3 g/mol and (2) subjects with no Fabry nephropathy as shown by normal UACR (≤ 3 g/mol). To decrease albuminuria, angiotensin-converting enzyme inhibitors (ACEIs) were prescribed to the patients with UACR > 3 g/mol.

### 2.2. Small uEVs-Enrichment with Size Exclusion Chromatography (SEC) 

Previously optimized method by Sedej et al. was used for the isolation of uEVs [25]. Briefly, 8 mL of the urine sample was mixed with 2 mL of 5x PBS and 160 µL of 10 mM EDTA, loaded onto the AMICON^®^ Ultra-15 Centrifugal Filter Units (MilliporeSigma, Burlington, MA, USA) and concentrated by centrifugation at 4000× *g* for 10 min at 17 °C. Moreover, 1x PBS was added to the centrifuged samples to the final volume of 500 µL. For uEVs separation, qEV original 70 nm columns (Izon Science Europe SAS, Lyon, France) were used. The columns were warmed to room temperature and washed with 1x PBS. Samples were then loaded onto the columns. Twenty sequential fractions of 500 µL were eluted with 1x PBS and collected into low protein binding tubes. Based on the A_280_ chromatogram (Synergy 2; BioTek, Winooski, VT, USA), proteins started to elute in fraction 13 with most proteins eluting in fractions 16–20 [25]. Therefore, only fractions 5–12 were pooled and further concentrated using AMICON^®^ Ultra-4 Centrifugal Filter Units (MilliporeSigma, Burlington, MA, USA) by centrifugation at 4000× *g* for 6 min at 4 °C. A 5 µL aliquot of the sample was stored in a low protein binding tube at −20 °C for nanoparticle tracking analysis (NTA). To the rest of the sample, water was added to a final volume of 200 µL followed by 800 µL of QIAzol Lysis Reagent (Qiagen, Hilden, Germany). Then it was stored at −80 °C in a low DNA binding tube until the isolation of miRNAs. 

### 2.3. Quantification of uEVs with NTA

The uEV-enriched samples were diluted in dPBS according to the detection range (10–100 particles/frame) and analyzed using a NanoSight NS300 (NanoSight Ltd., Malvern, UK) equipped with blue laser (488 nm, 45 mW) and sCMOS camera. Automatic settings for maximum jump distance, minimum expected particle size, and blur were selected. Minimum track length was set to 10, camera level to 15, detection threshold to 5, syringe pump flow to 20, and temperature to 25 °C. Five 60-s videos were recorded for each sample and the best three were selected for analysis after visual inspection. NTA software (version 3.4; NanoSight Ltd., Malvern, UK) was used to analyze the raw data of laser scattering and particle movement. The output data was presented as EV concentration (number of EVs enriched from 8 mL urine in particles/mL of urine) and EV size (hydrodynamic diameter in nm). 

### 2.4. Selection of miRNAs

We evaluated the expression of five uEV-derived miRNAs. In addition, two miRNAs were used for normalization. One of the most commonly used endogenous reference genes is RNU6B. However, this non-coding RNA is present only in nuclei and should not be detected in EVs or in fractions of circulating miRNAs [26]. Therefore, we searched the literature to find promising candidates. miR-16-5p [26] and miR-191-5p [27] were reported to be suitable candidates for normalization in patients with chronic and diabetic kidney disease (DKD) and were used for normalization of the expression of miRNAs in our study.

We selected five miRNAs that have been previously associated with different pathophysiological processes in kidney diseases. Urinary exosomal expression of miR-30b-5p was found to be downregulated in elderly patients with DKD. The levels also positively correlated with eGFR [28]. Members of miR-30 family may represent markers of glomerular disease and podocyte injury [29]. Its overexpression may have a protective role in podocyte injury [30] and in renal epithelial-to-mesenchymal transition [31]. miR-34a-5p was found upregulated in urine pellets and urine exosomes in DKD patients. Additionally, the expression of this miRNA positively correlated with serum creatinine and urinary protein-to-creatinine ratio (UPCR) [32]. miRNA-34a-5p was reported to promote renal fibrosis and epithelial-to-mesenchymal transition in tubular epithelial cells [33]. miR-23a-3p, miR-29a-3p and miR-200a-3p are implicated in renal fibrosis [34,35]. miR-29a-3p and miR-200a-3p are negatively regulated by transforming growth factor (TGF)-β and have anti-fibrotic effect by reducing the deposition of extracellular matrix and inhibiting epithelial-to-mesenchymal transition, respectively [35]. 

### 2.5. Quantification of uEV-Derived miRNAs with qPCR

Total RNA from EV preparations was isolated using the miRNeasy Mini Kit (Qiagen, Hilden, Germany) according to the manufacturer’s instructions. The protocol was adapted by the addition of extra 600 µL of RNase/DNase free water and subsequent chloroform extraction after the first removal of aqueous phase from the chloroform-sample mixture. RNA samples were eluted by two sequential additions of 25 μL of RNase/DNase free water. RNA quality and concentration were determined using NanoDrop One (Thermo Fisher Scientific, Waltham, MA, USA) and then RiboLock RNase Inhibitor (Thermo Fisher Scientific, Waltham, MA, USA) was added to each sample to prevent RNA degradation. Samples were stored at −80 °C in DNA low binding tubes (Eppendorf, Hamburg, Germany). 

Reverse transcription of miRNA into cDNA was performed according to the instructions of the miRCURY Locked Nucleic Acid (LNA) Universal RT kit (Qiagen, Hilden, Germany) using the GeneAmp^®^ PCR System 9700 (Applied Biosystems, Waltham, MA, USA). Reagents included 2 µL of 5x miRCURY RT SYBR^®^ Green Reaction Buffer, 4 µL of RNase free water, 1 µL of 10x miRCURY RT Enzyme and 3 µL of miRNA template. Reactions were performed at 42 °C for 60 min followed by 95 °C for 5 min. cDNA was stored at −20 °C in a low DNA binding plate (Eppendorf, Hamburg, Germany). 

miRNA expression analyses were performed by qPCR using 7 specific miRCURY LNA miRNA PCR Assays (Qiagen, Hilden, Germany) (Table 1). Samples were measured in triplicates along with a no template control. cDNA was diluted 1:30 by adding RNase-free water immediately before use. The reaction included 5 µL of 2x miRCURY SYBR^®^ Green Master Mix with added low ROX Reference Dye, 1 µL of resuspended PCR primer mix, 1 µL of RNase-free water, and 3 µL of cDNA template. Amplification was performed on the QuantStudio 7 Flex Real-Time PCR System (Applied Biosystems, Waltham, MA, USA) under the following conditions: 95 °C for 2 min, 40 cycles of 95 °C for 10 s and 56 °C for 1 min. The signal was collected at the endpoint of every cycle. Following amplification, melt curve analysis was performed to verify specificity. Ramping rate was set to 0.05 °C/s for 60–95 °C. 

The miRNA C_t_ values were normalized using the following formula: ΔC_t_ = average C_t_ (miRNA)–average C_t_ (reference miRNAs). The results were expressed as fold changes according to the equation 2^−^^ΔCt^ [36]. 

### 2.6. Statistical Analysis

All analyses were performed using IBM SPSS Statistic version 27.0 (IBM Corporation, New York, NY, USA). Categorical and continuous variables were described using frequencies and the median with interquartile (25–75%) range, respectively. A nonparametric Independent-Samples Mann-Whitney test was used to compare the distribution of continuous variables among patients with and without nephropathy. ANCOVA test was used for adjustment for clinical variables. A Wilcoxon signed-rank test was used for the comparison of chronological samples. Chi-Square and Fisher’s exact tests were used for categorical variables. Spearman’s Rho correlation coefficient was used to assess correlations between continuous variables. All statistical tests were two sided with the level of significance set to 0.05. 

## 3. Results

### 3.1. Patients Characteristics

The phenotypes of FD patients included in the study are shown in Table 2 together with the genetic variant in *GLA* gene. The characteristics of 20 included patients (12 females and 8 males) at last follow-up in 2020 are shown in Table 3. Fabry nephropathy was present in 11 (55%) FD patients, more specifically in seven females and four males. The median age of patients with nephropathy was significantly higher than the age of patients with normal renal function (*p* = 0.001). The median age at diagnosis of nephropathy based on UACR was 46 (36–60) years, while the median duration of nephropathy at last follow-up was 11 (4–12) years. All male patients and six female patients were treated with disease specific therapy (DST; ERT or chaperones). All patients with Fabry nephropathy except one (due to personal reasons) were taking ACEIs. One patient without Fabry nephropathy, but with a long history of diabetes, was also taking ACEI. There were statistically significant differences in UPCR, UACR, and eGFR between patients with and without nephropathy (all *p* = 0.001). Patients with Fabry nephropathy had higher UPCR and UACR and lower eGFR compared with patients without nephropathy. Patients with Fabry nephropathy also had higher daily proteinuria and steeper GFR decline, but the differences did not reach statistical significance (*p* = 0.067 and *p* = 0.080, respectively). There were also no significant differences in serum and urinary creatinine.

### 3.2. Similar Concentration and Size of uEVs in Patients without and with Fabry Nephropathy

The uEV characteristics at the last follow-up were compared between patients with and without Fabry nephropathy, using Independent-Sample Mann–Whitney test. No statistically significant differences in the concentration and sizes of uEVs were found between the groups (Table 4). In both groups, the modal diameters were slightly smaller compared to the mean and median diameters.

### 3.3. Positive Correlation between uEV Concentrations and Urinary Creatinine

The associations between the characteristics of uEVs and clinical parameters (Tables S1 and S2) were evaluated using Spearman’s correlation. A significant positive correlation was found between uEVs concentration and urinary creatinine in patients without and with Fabry nephropathy (ρ = 0.929, *p* < 0.001 and ρ = 0.636, *p* = 0.035, respectively). A positive correlation was found between mean diameter and eGFR (ρ = 0.627, *p* = 0.039) only in patients with Fabry nephropathy. No statistically significant correlations were found between other clinical parameters and uEVs characteristics.

Furthermore, ANCOVA test was performed to test if there was an overall statistically significant difference in uEVs concentration between the patients with and without Fabry nephropathy after adjustment for clinical parameters. There was no significant effect of nephropathy on uEVs concentration after adjustment for urinary creatinine (F = 2.985, *p* = 0.103). Therefore, we can conclude that urinary creatinine concentration has larger impact on uEVs concentration than the presence of nephropathy.

Due to the positive correlation between uEVs concentration and urinary creatinine in both groups, uEVs concentration was normalized to urinary creatinine and compared between patients with and without nephropathy. Nevertheless, even after normalization, there was no statistically significant difference in the ratio of uEVs concentration to urinary creatinine between patients without and with Fabry nephropathy (*p* = 0.310) at last follow-up.

### 3.4. No Difference in the Characteristics of uEVs between Chronological Samples

To test the hypothesis whether the presence and progression of nephropathy influence the characteristics of uEVs, Wilcoxon Signed Rank Test was used to assess changes in the concentration and sizes of uEVs between chronological samples. The results showed no statistically significant differences in the concentration and sizes of uEVs between samples collected either approximately 5 or 10 years apart (Table 5). Even when the analysis was performed separately for patients without and with Fabry nephropathy, there were no statistically significant differences, as shown in Tables S3 and S4.

### 3.5. Similar Expressions of uEV-Derived miRNAs in Patients without and with Fabry Nephropathy

We found no statistically significant differences in the expression of miRNAs between patients without and with Fabry nephropathy at the last follow-up in 2020, as shown in Table 6.

The results of the Spearman’s test for the correlation between the expression of miRNAs and clinical parameters are shown in Tables S5 and S6. A significant negative correlation was observed between miR-29a-3p and serum creatinine (ρ = −0.618, *p* = 0.043), but only in patients with Fabry nephropathy. Significant positive correlation was also observed between the duration of nephropathy and the expression of miR-29a-3p (ρ = 0.624, *p* = 0.040) and miR-30b-5p (ρ = 0.743, *p* = 0.009) in patients with Fabry nephropathy.

### 3.6. Changed Expression of miR-29a-3p, miR-200a-3p, and miR-30b-5p in Chronological Samples

To assess time-dependent changes in the expression of miRNAs, chronological samples were analyzed. Analysis of chronological samples collected approximately 5 years apart (*n* = 18) showed significantly different expression of miRNA-29a-3p and miRNA-200a-3p (*p* = 0.016 and *p* = 0.035, respectively). After separate analysis for the patients without and with Fabry nephropathy (Table 7), the difference in patients without nephropathy was no longer significant (*p* = 0.401 and *p* = 0.263, respectively), whereas the difference in the expression of miR-29a-3p in the patients with nephropathy remained significant (*p* = 0.017), and the expression of miR-200a-3p was borderline significant (*p* = 0.051). In the 5-year period, the expression of miR-29a-3p increased in 8 out of 10 FD patients while the expression of miR-200a-3p increased in 6 out of 9 patients with Fabry nephropathy.

In a cohort of 14 patients, we examined the changes in the expression of miRNAs in the samples collected approximately 10 years apart. In addition to miR-29a-3p and miR-200a-3p (both *p* = 0.001), the expression of miRNA-30b-5p was also significantly different (*p* = 0.048). However, in contrast to samples collected 5 years apart, the differences in the expression of miR-29a-3p and miR-200a-3p were significant in patients with as well as without Fabry nephropathy. On the other hand, the difference in expression of miR-30b-5p was significant only in patients without nephropathy (*p* = 0.028) and not in patients with Fabry nephropathy. Data are shown in Table 8. Interestingly, in all patients the expression of miR-29a-3p and miR-200a-3p increased over the 10-year period. The expression of miR-30b-5p decreased in two patients with Fabry nephropathy, while in other 12 patients without and with Fabry nephropathy the expression increased.

## 4. Discussion

FD patients with renal involvement show albuminuria, proteinuria, and reduced renal function [3]. In our cohort, patients with Fabry nephropathy differ significantly in UACR, UPCR, and eGFR from patients with no signs of renal impairment. However, these are all late markers of renal disease, indicating the need for discovery of new biomarkers that could allow early identification of at-risk individuals, which is of utmost importance for efficient treatment and improved prognosis. EVs and their molecular cargo, especially miRNAs, have been extensively studied and have shown great promise for identifying such biomarkers [13,38]. The present study is the first to characterize uEVs and uEV-derived miRNAs in chronological samples from FD patients.

Reported data on how progression of renal disease affects the excretion of uEVs are inconclusive. In patients with type 2 diabetes, it has been reported that the concentration of uEVs increases with the progression of albuminuria [39]. In contrast, no change in the total concentration and size of uEVs was observed in patients with type 1 diabetes [40]. Therefore, we investigated whether the concentration and sizes of total uEVs, isolated with SEC, could be considered as potential biomarkers of Fabry nephropathy. Our results suggest that total concentration and size are not indicative of either the presence or progression of Fabry nephropathy. In healthy control subjects, the number of uEVs was reported to be higher in younger subjects [41]. Specific subpopulations of uEVs or the relationship between them may better reflect pathophysiological changes within the kidney than total uEVs [42]. In FD, podocyte-derived uEVs would be of particular interest, as podocyte damage is one of the hallmarks of Fabry nephropathy, which was demonstrated in FD children with normal renal function [43]. Podocyte-derived uEVs represent only a small fraction of uEVs [44]. It is also important to emphasize that the lower detection limit of conventional flow cytometry is approximately 300 nm [45]; therefore, a nano flow cytometer should be used to detect specific markers of podocyte-derived small uEVs. Most studies described below assessed microparticles and not small uEVs as our study. In diabetic mice, increased secretion of podocyte-derived uEVs was detected as early as in the pre-albuminuria state, suggesting that this may be an early sign of podocyte damage [44]. Lytvyn et al. reported a significant increase in podocyte-derived uEVs in patients with type 1 diabetes compared to healthy controls, although no difference was observed in total concentration of uEVs. Moreover, the increase was seen in advance of albuminuria, nephrinuria, and GFR decline [40]. In the future, the study of specific uEVs populations in FD patients may provide more information on disease development and progression. On the other hand, secretion of uEVs may be impaired in advanced renal impairment, as shown by Kamińska et al. Namely, diabetic patients without renal failure had higher uEVs concentration compared to patients with renal failure [46]. However, if the excretion of uEVs changes only in the last stage of renal dysfunction, the concentration of uEVs cannot be considered as an early biomarker. Additionally, the comparison of the absolute concentrations and sizes of uEVs between different studies is hampered due to the lack of standardization in methodologies for isolation and studying of EVs [47].

Studies investigating the expression of miRNAs in FD patients are sparse. To our knowledge, only one study investigated the expression of miRNAs in the urine of FD patients in association with nephropathy [48]. They examined the expression of miRNAs in the urine sediment of young male FD patients with mild or absent nephropathy and in healthy subjects. Decreased expression of miR-29a-3p or/and miR-200a-3p was found in 4 out of 12 FD patients [48]. They also reported decreased expression of both miRNAs in a case report of a young male patient with classic FD [49]. However, we found no significant differences in the expression of selected uEV-derived miRNAs between FD patients with and without Fabry nephropathy at last follow-up, including the expression of miR-29a-3p and miR-200a-3p. Nevertheless, we found a significant difference in the expression of these two miRNAs in the chronological samples. Interestingly, the expression increased in the 5- and 10-year period in patients with Fabry nephropathy and in patients without nephropathy in the 10-year period. Pezzolesi et al., who also performed a longitudinal study, reported that one standard deviation increase in plasma expression miR-29a-3p was associated with protection against rapid progression to end-stage renal disease in patients with type 1 diabetes [50]. Since both miRNAs have similar roles in protection against fibrosis, the increase of miRNA-29a-3p and miR-200a-3p also in FD patients may indicate that there are some mechanisms trying to counteract the progression of renal dysfunction. It was previously shown that miR-29a-3p and miR-200a-3p are not associated with age and gender [51]. In addition, the expression of miR-30b-5p increased over the 10-year period, but only in FD patients without nephropathy. A previous study showed no change in miR-30b-5p expression between young and old control subjects [41]. Although the role of miR-30b-5p in the kidney was only recently discovered, the expression of miR-30b-5p is thought to have a protective role in podocyte injury [30] and endothelial-to-mesenchymal transition [31], which are both important stages in the development of nephropathy in FD. Since glycosphingolipid also accumulation occurs in patients without nephropathy, and at least some of the patients will develop renal dysfunction in the future, this may again indicate an attempt to ameliorate renal injury. To confirm these associations in FD, further studies are needed to investigate the molecular mechanisms associated with Fabry nephropathy. It should also be considered that the isolation protocol might influence the level of miRNAs [52]. 

The following limitations of the study should be considered. The cohort included in the study was relatively small; however, as FD is a rare disease, small groups are a common limitation of studies in FD. Furthermore, the impact of prolonged storage on the stability of EVs is not fully understood and requires further research [53]. Next, the study was limited to only five different candidate miRNAs and two reference miRNAs. Further studies using different approaches to evaluate the expression of a large number of miRNAs, such as microarrays, are needed to discover the combination of miRNAs that are most important for the development and progression of Fabry nephropathy. In addition, healthy control samples were not included in the study because chronological age-matched urine samples from healthy individuals were not available. However, it would also be interesting to investigate whether a difference between FD patients and healthy controls exists, and how the treatment, such as DST, ACEIs, statins, etc., affect the expression of uEV-derived miRNAs. Additionally, gender-specific miRNA analyses are needed to identify optimal biomarker candidates for Fabry nephropathy development and progression [54], as females present a more heterogeneous clinical picture with a wide range of disease severity due to random X chromosome inactivation [3].

## 5. Conclusions

uEVs could be used as a source of biomarkers of Fabry nephropathy because they are readily accessible and derived from the cells lining the urinary tract. Therefore, it is reasonable to expect them to reflect early changes in renal function. Our proof of concept study showed that EVs are abundant in the urine of FD patients and are applicable in studies of studies to predict the development and progression of nephropathy in FD. However, there was no difference in uEV characteristics between patients without and with Fabry nephropathy. Moreover, uEVs characteristics did not differ longitudinally. Therefore, the total concentration and size of uEVs were not indicative of either the presence or progression of Fabry nephropathy. Analysis of uEV-derived miRNAs showed no difference in the expression of five selected miRNAs. However, we found increased expression of miR-29a-3p and miR-200a-3p in chronological samples from patients with Fabry nephropathy, which may indicate an attempt by the organism to prevent the progression of renal damage leading to end-stage renal disease. In addition, we found an increased expression of miR-30b-5p in the 10-year period in FD patients without renal dysfunction, which may have a protective role in the development of nephropathy. Further studies with larger cohorts are needed to investigate the characteristics of the uEV subpopulations and the expression of a larger number of miRNAs to fully elucidate the complex pathophysiological processes in Fabry nephropathy. Since no difference in the characteristic of uEVs was found, the molecular cargo of uEVs may be more promising for the discovery of new biomarkers—not only transcriptomics analysis, but also other omics technologies, such as proteomics and metabolomics.

## Figures and Tables

**Table 1:** Selected miRNAs, sequences, and their accession numbers by Qiagen, Hilden, Germany.

miRNA	Sequence	Accession Number
hsa-miR-16-5p	5’UAGCAGCACGUAAAUAUUGGCG	MIMAT0000069
hsa-miR-23a-3p	5’UGGCUCAGUUCAGCAGGAACAG	MIMAT0000078
hsa-miR-29a-3p	5’UAGCACCAUCUGAAAUCGGUUA	MIMAT0000086
hsa-miR-30b-5p	5’UGUAAACAUCCUACACUCAGCU	MIMAT0000420
hsa-miR-34a-5p	5’UGGCAGUGUCUUAGCUGGUUGU	MIMAT0000255
hsa-miR-191-5p	5’CAACGGAAUCCCAAAAGCAGCUG	MIMAT0000440
hsa-miR-200a-3p	5’UAACACUGUCUGGUAACGAUGU	MIMAT0000682

**Table 2:** Genetic variants in *GLA* gene (reference sequence NM_000169.2) and phenotypes.

Patient	Genetic Variant		Nephropathy	HCMP	Stroke	Characteristic FD Symptoms
1	p.Arg363Pro	c.1088G>C	Yes	Yes	No	Yes
2	p.Asn272Ser	c.815A>G	No	Yes	No	Yes
3	p.Asn272Ser	c.815A>G	No	No	No	Yes
4	p.Asn272Ser	c.815A>G	Yes	Yes	No	Yes
5	p.Asn272Ser	c.815A>G	Yes	Yes	No	Yes
6	p.Asn272Ser	c.815A>G	No	Yes	No	Yes
7	p.Asn272Ser	c.815A>G	No	No	No	Yes
8	p.Asn272Ser	c.815A>G	No	No	No	No
9	p.Asn272Ser	c.815A>G	No	No	No	Yes
10	p.Glu358del	c.1072_1074delGAG	Yes	Yes	Yes	Yes
11	p.Glu87Asp	c.261_278del18	Yes	No	No	Yes
12	p.Arg342Gln	c.1025G>A	Yes	Yes	No	Yes
13	p.Ile270Met	c.810T>G	Yes	Yes	Yes	Yes
14	p.Ile270Met	c.810T>G	Yes	Yes	No	Yes
15	p.Arg227Ter	c.679C>T	No	No	No	Yes
16	p.Leu180Phe	c.540G>C	Yes	Yes	Yes	Yes
17	p.Leu180Phe	c.540G>C	No	No	No	No
18	p.Gly261ValfsTer8	c.789delG	Yes	Yes	Yes	Yes
19	p.Gly261ValfsTer8	c.789delG	No	No	No	Yes
20	p.Tyr152His	c.454T>C	Yes	Yes	No	No

Nephropathy was defined as albuminuria with urinary albumin-to-creatinine ratio (UACR) > 3 g/mol. Hypertrophic cardiomyopathy (HCMP) was assessed by echocardiography and/or cardiac MRI and defined according to the literature [37]. Stroke was confirmed by appropriate imaging assessment. Characteristic Fabry disease (FD) symptoms were defined when present Fabry neuropathic pain, angiokeratoma, and/or cornea verticillata.

**Table 3:** Patients’ characteristics at last follow-up (*n* = 20).

Parameter		Absolute Frequency (%)		*p* Value
		No Nephropathy	Nephropathy	
Gender	Male	4 (20)	4 (20)	0.714
	Female	5 (25)	7 (35)	
DST	No	4 (20)	2 (10)	0.336
	Yes	5 (25)	9 (45)	
ACEI	No	8 (40)	1 (5)	**0.001**
	Yes	1 (5)	10 (50)	
Diabetes	No	8 (40)	8 (40)	0.591
	Yes	1 (5)	3 (15)	
**Parameter**		**Median (25–75%)**		***p* Value**
		**No Nephropathy**	**Nephropathy**	
Age (years)		32.0 (27.3–48.8)	58.9 (47.9–65.8)	**0.001**
UPCR (g/mol)		9.60 (8.25–10.9)	32.2 (12.2–95.2) *	**0.001**
UACR (g/mol)		0.85 (0.60–1.55) *	7.80 (2.35–81.5) **	**0.001**
D-prot (g/day)		0.11 (0.09–0.16)	0.20 (0.14–1.41)	0.067
S-Cr (µmol/L)		63.0 (55.5–72.5)	80.0 (58.0–87.0)	0.175
U-Cr (µmol/L)		5489 (3180–9601) *	5441 (3247–10,802)	0.657
eGFR (mL/min/1.73 m^2^)		116.0 (99.5–119.5)	81.0 (64.0–95.0)	**0.001**
GFR slope (mL/min/year)		−0.71 (−2.30–1.87)	−2.17 (−4.34–−1.28)	0.080

DST, disease specific therapy; ACEI, angiotensin-converting enzyme inhibitor; UPCR, urinary protein-to-creatinine ratio; UACR, urinary albumin-to-creatinine ratio; D-prot, daily proteinuria; S-Cr, serum creatinine; U-Cr, urinary creatinine; eGFR, estimated glomerular filtration rate; GFR, glomerular filtration rate. Bold font indicates statistical significance. * One datum is missing. ** Two data are missing.

**Table 4:** Medians of concentration, mean, modal, and median diameter in FD patients without (*n* = 9) and with (*n* = 11) nephropathy at last follow-up.

Parameter	Median (25–75%)		*p* Value
	No Nephropathy	Nephropathy	
Concentration (× 10^11^/mL)	3.26 (1.23–11.7)	1.89 (1.27–9.72)	0.766
Mean Diameter (nm)	166.4 (140.5–173.0)	161.8 (140.5–176.5)	0.882
Modal Diameter (nm)	121.3 (109.4–146.0)	113.8 (96.1–129.6)	0.370
Median Diameter (nm)	150.8 (126.3–157.5)	145.5 (129.8–161.4)	0.941

**Table 5:** uEV characteristics of chronological samples in 5- (*n* = 18) and 10-year periods (*n* = 14).

Parameter	Median (25–75%)		*p* Value
	5 Years Ago	Last Follow-up	
Concentration (× 10^11^/mL)	4.34 (1.90–7.77)	1.96 (1.26–9.42)	0.215
Mean Diameter (nm)	158.1 (148.1–165.3)	164.1 (140.5–173.7)	0.446
Modal Diameter (nm)	120.9 (113.2–132.2)	115.3 (105.9–136.3)	0.777
Median Diameter (nm)	143.3 (132.5–153.0)	148.2 (129.4–156.8)	0.528
**Parameter**	**Median (25–75%)**		***p* Value**
	**10 Years Ago**	**Last Follow-up**	
Concentration (× 10^11^/mL)	7.21 (5.43–17.6)	2.63 (1.26–9.42)	0.096
Mean Diameter (nm)	152.8 (143.8–162.4)	160.6 (139.1–172.8)	0.754
Modal Diameter (nm)	113.7 (107.8–120.3)	115.3 (105.9–130.8)	0.900
Median Diameter (nm)	135.3 (129.4–147.4)	142.8 (127.8–156.2)	0.551

**Table 6:** Fold changes in miRNAs expression in patients without (*n* = 9) and with (*n* = 11) Fabry nephropathy at last follow-up.

Parameter	Median (25–75%)		*p* Value
	No Nephropathy	Nephropathy	
hsa-miR-200a-3p	2.73 (1.45–3.82)	2.35 (1.79–3.34) *	0.905
hsa-miR-29a-3p	1.05 (0.91–2.58)	1.06 (0.85–2.88)	0.766
hsa-miR-30b-5p	7.11 (6.42–10.7)	7.81 (5.39–10.4)	0.603
hsa-miR-23a-3p	1.66 (0.76–2.19)	1.30 (0.96–1.68)	0.766
hsa-miR-34a-5p	0.14 (0.10–0.19)	0.16 (0.06–0.23)	0.941

* One datum is missing.

**Table 7:** Fold changes in the expression of miRNAs in (a) all patients (*n* = 18), (b) patients without nephropathy (*n* = 8), and (c) patients with Fabry nephropathy (*n* = 10) in samples collected approximately 5 years apart.

(a) All Patients	**Parameter**	**Median (25–75%)**		***p* Value**
		**5 Years Ago**	**Last Follow-up**	
	hsa-miR-200a-3p	1.96 (1.48–2.25)	2.73 (1.71–3.69) *	**0.035**
	hsa-miR-29a-3p	0.81 (0.55–1.10)	1.06 (0.92–2.76)	**0.016**
	hsa-miR-30b-5p	7.62 (4.88–12.1)	7.47 (6.48–10.4)	0.711
	hsa-miR-23a-3p	2.10 (1.45–2.81)	1.38 (0.92–2.17)	0.184
	hsa-miR-34a-5p	0.13 (0.10–0.19)	0.14 (0.09–0.20)	0.777
(b) Without Nephropathy	**Parameter**	**Median (25–75%)**		***p* Value**
		**5 Years Ago**	**Last Follow-up**	
	hsa-miR-200a-3p	2.02 (1.15–2.71)	2.77 (1.63–4.09)	0.263
	hsa-miR-29a-3p	0.92 (0.82–1.82)	1.04 (0.90–2.61)	0.401
	hsa-miR-30b-5p	9.67 (6.44–14.9)	7.04 (6.36–10.3)	0.484
	hsa-miR-23a-3p	2.43 (1.79–3.38)	1.76 (0.86–2.20)	0.484
	hsa-miR-34a-5p	0.11 (0.08–0.23)	0.13 (0.10–0.18)	1.000
(c) With Nephropathy	**Parameter**	**Median (25–75%)**		***p* Value**
		**5 Years Ago**	**Last Follow-up**	
	hsa-miR-200a-3p	1.96 (1.48–2.16)	2.58 (1.71–3.59) *	0.051
	hsa-miR-29a-3p	0.60 (0.48–0.89)	1.11 (0.94–2.94)	**0.017**
	hsa-miR-30b-5p	6.40 (4.50–8.65)	8.37 (6.26–10.5)	0.333
	hsa-miR-23a-3p	1.87 (1.39–2.45)	1.29 (0.89–1.86)	0.169
	hsa-miR-34a-5p	0.14 (0.11–0.19)	0.15 (0.06–0.25)	0.721

Bold font indicates statistical significance. * One datum is missing.

**Table 8:** Fold changes in the expression of miRNAs in (a) all patients (*n* = 14), (b) patients without nephropathy (*n* = 6), and (c) patients with Fabry nephropathy (*n* = 8) in samples collected approximately 10 years apart.

(a) All Patients	**Parameter**	**Median (25–75%)**		***p* Value**
		**10 Years Ago**	**Last Follow-up**	
	hsa-miR-200a-3p	1.49 (1.36–2.02)	2.81 (1.99–4.13) *	**0.001**
	hsa-miR-29a-3p	0.66 (0.34–0.84)	1.79 (0.98–2.88)	**0.001**
	hsa-miR-30b-5p	5.19 (3.59–11.2)	9.29 (6.48–10.6)	**0.048**
	hsa-miR-23a-3p	1.55 (1.05–1.93)	1.29 (0.79–1.78)	0.510
	hsa-miR-34a-5p	0.17 (0.10–0.27)	0.12 (0.07–0.19)	0.826
(b) Without Nephropathy	**Parameter**	**Median (25–75%)**		***p* Value**
		**10 Years Ago**	**Last Follow-up**	
	hsa-miR-200a-3p	1.54 (1.33–1.95)	3.05 (2.21–4.76)	**0.028**
	hsa-miR-29a-3p	0.71 (0.25–1.00)	1.78 (1.00–2.70)	**0.028**
	hsa-miR-30b-5p	7.07 (3.35–10.0)	8.31 (6.49–11.7)	**0.028**
	hsa-miR-23a-3p	1.58 (0.93–1.70)	1.34 (0.79–2.17)	0.917
	hsa-miR-34a-5p	0.12 (0.08–0.27)	0.12 (0.09–0.22)	0.600
(c) With Nephropathy	**Parameter**	**Median (25–75%)**		***p* Value**
		**10 Years Ago**	**Last Follow-up**	
	hsa-miR-200a-3p	1.49 (1.35–2.18)	2.58 (1.86–4.09) *	**0.018**
	hsa-miR-29a-3p	0.60 (0.41–0.88)	1.89 (0.97–3.08)	**0.012**
	hsa-miR-30b-5p	5.02 (3.88–13.0)	9.63 (5.68–10.7)	0.401
	hsa-miR-23a-3p	1.32 (1.07–2.09)	1.29 (0.76–1.58)	0.161
	hsa-miR-34a-5p	0.21 (0.10–0.35)	0.12 (0.05–0.22)	0.575

Bold font indicates statistical significance. * One datum is missing.

## Data Availability

The data presented in this study are available upon request from the corresponding author.

## Supplementary Materials

The following are available online at https://www.mdpi.com/article/10.3390/genes12071057/s1, Table S1: Spearman’s correlation analysis of uEVs characteristics and clinical parameters in FD patients without nephropathy (*n* = 9), Table S2: Spearman’s correlation analysis of uEVs characteristics and clinical parameters in FD patients with nephropathy (*n* = 10), Table S3: Medians of concentration, mean, modal, and median diameter in patients (a) without (*n* = 8) and (b) with Fabry nephropathy (*n* = 10) in 5-year period, Table S4: Medians of concentration, mean, modal, and median diameter in patients (a) without (*n* = 6) and (b) with Fabry nephropathy (*n* = 8) in 10-year period, Table S5: Spearman’s correlation analysis between expression of miRNAs and clinical parameters in FD patients without nephropathy (*n* = 9), Table S6: Spearman’s correlation analysis between expression of miRNAs and clinical parameters in FD patients with nephropathy (*n* = 10).

## Abbreviations

α-gal A	α-galactosidase A
ACEI	angiotensin-converting enzyme inhibitor
D-prot	daily proteinuria
DKD	diabetic kidney disease
DST	disease specific therapy
eGFR	estimated glomerular filtration rate
EVs	extracellular vesicles
FD	Fabry disease
GFR	glomerular filtration rate
miRNA, miR	microRNA
NTA	nanoparticle tracking analysis
S-Cr	serum creatinine
SEC	size exclusion chromatography
UACR	urinary albumin-to-creatinine ratio
U-Cr	urinary creatinine
uEVs	urinary extracellular vesicles
UPCR	urinary protein-to-creatinine ratio
