# Supplementary material for: Urinary Extracellular Vesicles and Their miRNA Cargo in Patients with Fabry Nephropathy
Source: Genes (Basel). 2021 Jul 9;12(7):1057. doi: 10.3390/genes12071057 (PMC8305897; doi:10.3390/genes12071057)
Supplement: Supplementary file 1 [file genes-12-01057-s001.zip › genes-1245581-supplementary.pdf]

Supplementary Materials

Table S1. Spearman's correlation analysis of uEVs characteristics and clinical parameters in FD patients without nephropathy (n = 9).

Parameter	Concentration		Mean Diameter		Modal Diameter		Median Diameter	
	ϱ	*p* Value	ϱ	*p* Value	ϱ	*p* Value	ϱ	*p* Value
UPCR	-0.333	0.381	-0.317	0.406	0.050	0.898	-0.293	0.444
UACR	0.491	0.217	0.335	0.417	0.311	0.453	0.355	0.388
D-prot	-0.100	0.797	0.452	0.222	0.368	0.330	0.483	0.188
S-Cr	-0.226	0.559	0.184	0.635	0.075	0.847	0.147	0.706
U-Cr	0.929	**< 0.001**	0.667	0.071	0.310	0.456	0.611	0.108
eGFR	-0.430	0.248	-0.068	0.863	-0.135	0.729	-0.144	0.712
GFR slope	-0.100	0.798	-0.067	0.865	0.517	0.154	0.075	0.847

UPCR, urinary protein-to-creatinine ratio; UACR, urinary albumin-to-creatinine ratio; D-prot, daily proteinuria; S-Cr, serum creatinine; U-Cr, urinary creatinine; eGFR, estimated glomerular filtration rate; GFR, glomerular filtration rate.

Table S2. Spearman's correlation analysis of uEVs characteristics and clinical parameters in FD patients with nephropathy (n = 10).

Parameter	Concentration		Mean Diameter		Modal Diameter		Median Diameter	
	ϱ	*p* Value	ϱ	*p* Value	ϱ	*p* Value	ϱ	*p* Value
UPCR	-0.091	0.803	0.042	0.907	0.273	0.446	0.139	0.701
UACR	-0.050	0.898	0.317	0.406	0.333	0.381	0.333	0.381
D-prot	-0.146	0.668	0.160	0.639	0.205	0.544	0.251	0.456
S-Cr	-0.518	0.102	-0.600	0.051	-0.455	0.160	-0.555	0.077
U-Cr	0.636	**0.035**	0.509	0.110	0.282	0.401	0.382	0.247
eGFR	0.564	0.071	0.627	**0.039**	0.355	0.285	0.582	0.060
GFR slope	0.473	0.142	0.600	0.051	0.245	0.467	0.582	0.060
Duration	0.037	0.915	-0.284	0.397	-0.073	0.830	-0.220	0.515

UPCR, urinary protein-to-creatinine ratio; UACR, urinary albumin-to-creatinine ratio; D-prot, daily proteinuria; S-Cr, serum creatinine; U-Cr, urinary creatinine; eGFR, estimated glomerular filtration rate; GFR, glomerular filtration rate; duration, duration of nephropathy.

Table S3. Medians of concentration, mean, modal, and median diameter in patients (a) without (n = 8) and (b) with Fabry nephropathy (n = 10) in 5-year period.

	Parameter	Median (25–75%)		*p* Value
		5 Years Ago	Last Follow-up	
(a) Without Nephropathy	Concentration (× 10^{11}/mL)	3.22 (1.44–6.64)	3.66 (1.39–14.0)	0.889
	Mean Diameter (nm)	158.8 (151.8–186.8)	169.1 (141.3–173.2)	1.000
	Modal Diameter (nm)	125.2 (112.1–132.5)	117.2 (108.7–151.7)	0.889
	Median Diameter (nm)	143.2 (132.6–164.6)	153.5 (126.3–158.1)	0.779
	**Parameter**	**Median (25–75%)**		***p* Value**
		**5 Years Ago**	**Last Follow-up**	
(b) With Nephropathy	Concentration (× 10^{11}/mL)	6.53 (2.23–9.99)	1.75 (1.17–6.89)	0.093
	Mean Diameter (nm)	158.1 (145.3–162.2)	156.6 (140.5–175.2)	0.386
	Modal Diameter (nm)	119.7 (111.9–128.3)	115.3 (94.6–132.7)	0.712
	Median Diameter (nm)	143.5 (131.5–152.3)	142.3 (129.4–157.9)	0.575

Table S4. Medians of concentration, mean, modal, and median diameter in patients (a) without (n = 6) and (b) with Fabry nephropathy (n = 8) in 10-year period.

	Parameter	Median (25–75%)		*p* Value
		10 Years Ago	Last Follow-up	
(a) Without Nephropathy	Concentration (× 10^{11}/mL)	8.84 (5.51–18.1)	3.66 (1.10–9.42)	0.463
	Mean Diameter (nm)	157.3 (147.4–171.5)	162.9 (133.9–172.8)	0.753
	Modal Diameter (nm)	113.7 (108.4–130.6)	117.2 (106.3–140.2)	0.752
	Median Diameter (nm)	139.1 (132.8–154.4)	148.2 (120.4–156.8)	0.753
	**Parameter**	**Median (25–75%)**		***p* Value**
		**10 Years Ago**	**Last Follow-up**	
(b) With Nephropathy	Concentration (× 10^{11}/mL)	7.21 (4.19–18.4)	1.95 (1.36–13.9)	0.123
	Mean Diameter (nm)	150.3 (142.8–157.9)	156.6 (140.4–172.7)	0.484
	Modal Diameter (nm)	114.0 (106.2–120.5)	115.3 (98.9–126.9)	0.889
	Median Diameter (nm)	132.3 (129.3–146.0)	142.3 (130.0–155.7)	0.263

Table S5. Spearman's correlation analysis between expression of miRNAs and clinical parameters in FD patients without nephropathy (n = 9).

Parameter	miR-23a-3p		miR-29a-3p		miR-30b-5p		miR-34a-5p		miR-200a-3p	
	ϱ	*p* Value	ϱ	*p* Value	ϱ	*p* Value	ϱ	*p* Value	ϱ	*p* Value
UPCR	-0.450	0.224	0.350	0.356	0.200	0.606	-0.033	0.932	-0.150	0.700
UACR	-0.335	0.417	-0.192	0.649	0.036	0.933	-0.120	0.778	-0.563	0.146
D-prot	0.050	0.898	-0.218	0.574	0.360	0.342	-0.050	0.898	0.109	0.781
S-Cr	-0.259	0.500	-0.176	0.651	0.368	0.330	0.276	0.472	-0.184	0.635
U-Cr	0.381	0.352	-0.262	0.531	-0.667	0.071	-0.262	0.531	-0.262	0.531
eGFR	0.051	0.897	0.118	0.762	-0.059	0.880	-0.034	0.931	0.169	0.664
GFR slope	-0.233	0.546	0.233	0.546	0.183	0.637	-0.167	0.668	-0.283	0.460

UPCR, urinary protein-to-creatinine ratio; UACR, urinary albumin-to-creatinine ratio; D-prot, daily proteinuria; S-Cr, serum creatinine; U-Cr, urinary creatinine; eGFR, estimated glomerular filtration rate; GFR, glomerular filtration rate.

Table S6. Spearman's correlation analysis between expression of miRNAs and clinical parameters in FD patients with nephropathy (n = 10).

Parameter	miR-23a-3p		miR-29a-3p		miR-30b-5p		miR-34a-5p		miR-200a-3p	
	ϱ	*p* Value	ϱ	*p* Value	ϱ	*p* Value	ϱ	*p* Value	ϱ	*p* Value
UPCR	-0.382	0.276	0.285	0.425	0.552	0.098	-0.370	0.293	0.433	0.244
UACR	-0.267	0.488	0.050	0.898	0.467	0.205	-0.233	0.546	0.429	0.289
D-prot	-0.100	0.769	-0.160	0.639	0.009	0.979	0.055	0.873	0.347	0.327
S-Cr	0.245	0.467	-0.618	**0.043**	-0.545	0.083	0.418	0.201	0.006	0.987
U-Cr	0.309	0.355	0.045	0.894	-0.182	0.593	-0.073	0.832	-0.115	0.751
eGFR	-0.300	0.370	0.482	0.133	0.482	0.133	-0.327	0.326	-0.042	0.907
GFR slope	-0.255	0.450	0.155	0.650	0.300	0.370	-0.118	0.729	0.115	0.715
Duration	-0.266	0.429	0.624	**0.040**	0.743	**0.009**	-0.541	0.085	0.146	0.688

UPCR, urinary protein-to-creatinine ratio; UACR, urinary albumin-to-creatinine ratio; D-prot, daily proteinuria; S-Cr, serum creatinine; U-Cr, urinary creatinine; eGFR, estimated glomerular filtration rate; GFR, glomerular filtration rate; duration, duration of nephropathy.
